# Paradigms of receptor kinase signaling in plants

**DOI:** 10.1042/BCJ20220372

**Published:** 2023-06-16

**Authors:** Kyle W. Bender, Cyril Zipfel

**Affiliations:** 1 Institute of Plant and Microbial Biology, Zürich-Basel Plant Science Center, University of Zürich, 8008 Zürich, Switzerland; 2 The Sainsbury Laboratory, University of East Anglia, Norwich Research Park, NR4 7UH Norwich, U.K.

**Keywords:** receptor kinase, receptor-like cytoplasmic kinase, signaling

## Abstract

Plant receptor kinases (RKs) function as key plasma-membrane localized receptors in the perception of molecular ligands regulating development and environmental response. Through the perception of diverse ligands, RKs regulate various aspects throughout the plant life cycle from fertilization to seed set. Thirty years of research on plant RKs has generated a wealth of knowledge on how RKs perceive ligands and activate downstream signaling. In the present review, we synthesize this body of knowledge into five central paradigms of plant RK signaling: (1) RKs are encoded by expanded gene families, largely conserved throughout land plant evolution; (2) RKs perceive many different kinds of ligands through a range of ectodomain architectures; (3) RK complexes are typically activated by co-receptor recruitment; (4) post-translational modifications fulfill central roles in both the activation and attenuation of RK-mediated signaling; and, (5) RKs activate a common set of downstream signaling processes through receptor-like cytoplasmic kinases (RLCKs). For each of these paradigms, we discuss key illustrative examples and also highlight known exceptions. We conclude by presenting five critical gaps in our understanding of RK function.

## Introduction

Timely and appropriate cellular response to external signals is a central tenet in biology, required for diverse aspects of organism function ranging from, for example, the establishment of developmental patterns in metazoans to bacterial chemotaxis to immune system activation and regulation in plants. Cellular responses to external stimuli are coordinated by highly interconnected signal transduction cascades whose activation drives wide-spread adaptive reprogramming of cellular states (e.g. transcriptional landscapes, protein post-translational modifications, metabolite profiles). At the apex of signal transduction cascades lie proteins that function as receptors, capable of perceiving signals of both molecular and non-molecular origin and translating them into cellular action. Receptor proteins take on diverse architectures, allowing them to activate signaling events directly or indirectly through effector proteins. Additionally, receptors are organized broadly throughout the cell. While many receptors are resident in the plasma membrane where they perceive signals external to the cell, others reside in the cytosol or nucleus and perceive signals arriving in the cytosol through diverse mechanisms.

The most abundant class of cell surface receptors in eukaryotes are the metazoan G protein-coupled receptors [1]. Although present in some green algae — for example, the Channelrhodopsin of *Chlamydomonas reinhardtii* — the presence of *bona fide* GPCRs in land plants remains controversial [2,3]. Rather, a novel class of receptor proteins has diversified in the green lineage — the receptor kinases (RKs) [4,5]. RKs are plasma membrane (PM)-localized, enzyme-linked receptors with diverse ectodomain architectures that detect myriad different molecular signals [6]. Plant genomes encode hundreds of RKs, underscoring their importance for the establishment and regulation of plant form and function.

Early work in the model organism *Arabidopsis thaliana* (hereafter, Arabidopsis) demonstrated the central role of RKs in plant development. At the end of the 1990's, a plethora of genetic studies revealed roles for RKs in the perception of plant steroid hormones (brassinosteroids, BRs), the regulation of plant meristem maintenance, and plant morphogenesis [7–10]. Subsequently, RKs were implicated in mediating the interaction between plants and microorganisms [11–17]. The last three decades have positioned RK identification and functional characterization as a critical focus area in plant biology. In that time, several common themes have emerged with respect to the evolution and function of plant RKs. In the present review, we highlight five key areas of knowledge and five key knowledge gaps we should endeavor to fill for a holistic understanding of plant RK structure and function.

## Five paradigms of plant receptor kinase signaling

### RK architecture mirrors transmembrane signaling components from metazoans

**Figure 1.**
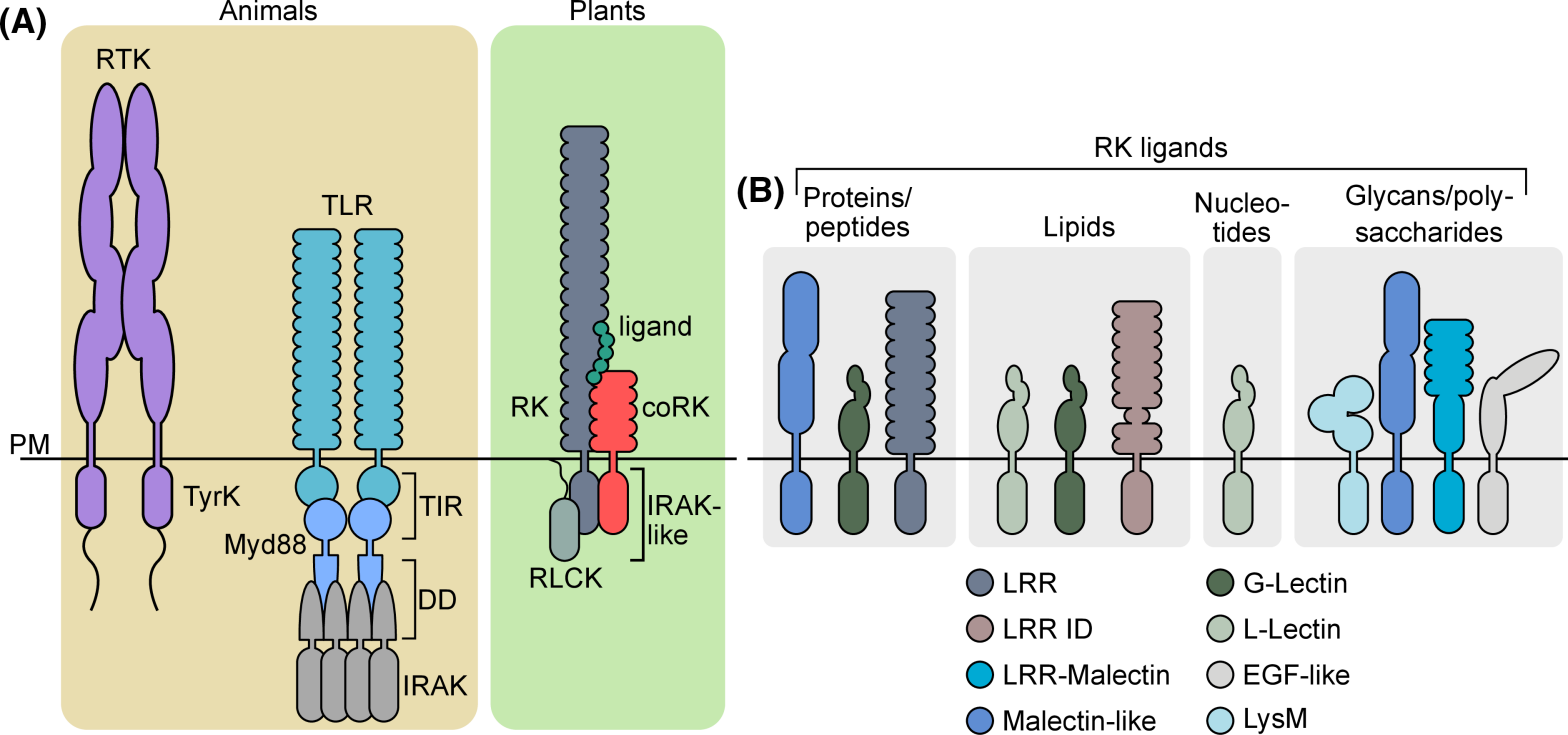
Architecture of selected transmembrane signaling proteins. (**A**) Comparison of selected single-pass transmembrane receptors from animals and plants. RTKs consist of ligand binding extracellular domains tethered to intracellular tyrosine kinase domains and have long C-terminal tails involved in the recruitment of signaling partners. Activated TLRs form a large multimeric complex called the Myddosome. The complex is nucleated by TIR and DD of the protein, Myd88. Myd88 DD recruits multiple IRAKs that execute downstream signaling. Plant RKs consist of extracellular ligand-binding domains directly tethered to a cytoplasmic IRAK-like protein kinase domain and typically function with a co-receptor (coRK) and RLCKs that activate downstream signaling. An LRR-RK complex is shown as an example. (**B**) Plant RKs have diverse ectodomains that perceive a broad range of endogenous and exogenous ligands.

Soon after the identification of the first RKs in plants, it was recognized that their overall structure mirrored that of the receptor tyrosine kinases (RTKs) from animals. Like RTKs, plant receptor kinases consist of an extracellular domain (ECD), a single-pass transmembrane domain, and an intracellular eukaryotic protein kinase (ePK) domain (Figure 1A). However, plant RKs carry ECDs with largely distinct architectures compared with animal RTKs [18]. While the ePK domains of plant RKs are phylogenetically related to RTKs, plant RKs are not the direct plant homologs of RTKs. Rather, the plant RKs form a monophyletic group with the metazoan Pelle/INTERLEUKIN-1 RECEPTOR ASSOCIATED KINASE (IRAKs) family of Ser/Thr protein kinases [4]. This family subsequently underwent considerable diversification in the lineage giving rise to the land plants, but remained rather restricted in metazoans [5,19,20].

In metazoans, Pelle/IRAK proteins function as key signal transmitters in innate immunity downstream of Toll-like receptors (TLRs). TLRs are a family of PM- or endosome-localized leucine-rich repeat (LRR) transmembrane proteins that perceive different pathogen-associated molecular patterns (PAMPs) from a range of microorganisms [21]. Rather than signaling directly via an enzymatically active cytoplasmic domain as is the case for RTKs and RKs, TLRs carry TOLL-INTERLEUKIN RECEPTOR (TIR) domains in the cytoplasm that dimerize upon ligand perception to initiate the formation of a supramolecular organizing complex called the Myddosome. TLR TIR domains are recognized by the TIR domains from additional proteins called TIRAP and MyD88. MyD88 additionally contains a so-called ‘death domain’ that subsequently interacts with the death domain of IRAKs [21]. These components assemble together in an oligomeric complex. The end result is a large helical complex containing several IRAK molecules whose protein kinase activities are only activated within the assembled Myddosome [21–23]. One can therefore consider plant RK complexes to be ‘all-in-one’ Myddosomes where ligand-perceiving ECDs are directly tethered to intracellular IRAK-like ePK domains that only become activated upon receptor complex formation (see Regulation of RK signaling by complex formation).

Owing to their diversification during land plant evolution, RKs are among the largest gene families in plants. For example, the model dicot *Arabidopsis thaliana* encodes for greater than 600 RKs and receptor-like cytoplasmic kinases (RLCKs) and rice (*Oryza sativa*) encodes for more than 1000. The genome of the early-diverging land plant *Marchantia polymorpha* contains greater than 200 RK/RLCK genes [19]. The RK family can be subdivided into nearly 60 subfamilies based on the relatedness of ePK domains, and nearly all of these families are already present in liverworts and mosses, suggesting an early origin for RK diversity during land plant evolution [6,24,25]. Large-scale phylogenetic analyses of plant RKs — in particular of the LRR-RK family — indicate that the bulk of diversity within the RK family derives from expansion within RK subfamilies, although not having undergone equivalent diversification. In particular, RK subfamilies implicated in immune function, for example the LRR-XIIa subfamily, has experienced substantial lineage-specific expansion coordinately with other plant immune system components [26,27]. Plant genomes also encode expanded families of receptor-like proteins (RLPs) [28–31]. RLPs represent a second class of plant transmembrane receptors but are distinct from the RKs in that they lack cytoplasmic protein kinase domains. Instead, RLPs function together with RKs having shared ECD architectures. While the topics discussed in the present review apply additionally to RLP-mediated signaling, the RLPs have been recently reviewed elsewhere and thus we do not focus on them here [29,31].

The plant RK family contains a diversity of ECD architectures that were mostly established during the evolution of land plants, although some architectures were acquired earlier [19]. That most known plant RK ECDs are present in liverworts and mosses suggests that the acquisition of novel ECDs is not a major path towards neofunctionalization within the RK family. Indeed, only relatively few domain-gain events have been detected for RKs in angiosperms [24]. One important exception is the gain of putative glycan-binding malectin-like domains in the ECDs of LRR-I and LRR-VIII-2 proteins [32]. Conversely, domain loss — of either the ECD or the cytoplasmic domain — has occurred more frequently, leading to substantial structural variation across the LRR-RK family. Such patterns of domain loss are observed in most LRR clades, and in some cases are deeply rooted in evolution. For example, several proteins consisting of only a short LRR domain were recently discovered as members of the LRR-II clade that contains RKs with co-receptor functions [32]. How such ECD-only proteins might contribute to ligand perception and or downstream signaling remains an open question.

In addition to functional diversification through domain reorganization, LRR-RK genes are, on average, under stronger positive selection compared with the rest of the genome; although such trends are not observed equally across the LRR-RK family [26,33,34]. As might be expected in the context of plant–pathogen interactions, LRR-RK families containing immune receptors (e.g. LRR-XII) tend to experience increased rates of positive selection compared with others, but developmental RKs (e.g. LRR-XI) also undergo positive selection [33,34]. Interestingly, selective pressures are accelerated to a greater extent for ECDs compared with cytoplasmic domains, highlighting the possibility for diversification of signaling inputs converging on similar intracellular signaling pathways [26,33,34]. Such evolutionary trends are also observed for non-LRR-RKs, suggesting that common evolutionary processes operate across the broader RK family [34].

### RK ECDs perceive myriad molecular signals

The architecture of plant RK ECDs underlies their capacity to detect a large repertoire of signals (Figure 1B), and much of our knowledge regarding the different types of biological macromolecules sensed by RKs comes from our understanding of plant–microbe interactions and the detection of PAMPs. In this context, many RKs function as pattern-recognition receptors (PRRs) that directly bind PAMPs, while others detect phytocytokines — endogenous peptides regulating immunity — or damage-associated molecular patterns (DAMPs) — self-derived molecules that are released during the interaction with microbes or upon wounding. The LRR-ECD PRRs FLAGELLIN SENSING 2 (FLS2) and ELONGATION FACTOR Tu RECEPTOR (EFR) perceive the conserved peptide epitopes flg22 and elf18 derived from bacterial flagellin and elongation factor thermo unstable, respectively. Interestingly, multiple distinct epitopes derived from the flagellin protein are perceived by different LRR-ECD PRRs across different plant species [35,36]. While the flg22 epitope is perceived broadly across the diversity of plants, other species such as tomato (*Solanum lycopersicum*) perceive an additional epitope known as flgII-28 [35]. Peptide epitopes are also perceived by PRRs in the context of interaction with parasitic plants as well as insects and nematodes [37].

In addition to peptides, several PAMPs derived from the cell walls of bacteria and fungi — peptidoglycan and chitin, respectively — are perceived by RKs, in this case with lysin motif (LysM) ECDs (LysM-RKs) [37]. LysM domains are broadly represented in the genomes of prokaryotes and eukaryotes and function in both glycan binding and hydrolysis in different contexts [38]. Interestingly, LysM-RKs are also involved in the perception of both rhizobial and mycorrhizal symbionts [39]. Chimeric receptor analysis and structural comparisons of LysM-RKs involved in symbiosis and immunity indicate that motifs in LysM1 (the first of three LysM domains in the ECD) underlie perception specificity for different chitinous ligands [40]. In addition to the perception of microbe-derived polysaccharides, plant cell wall derivatives are perceived as DAMPs during plant–microbe interactions. Oligogalacturonides (OG) originating from the homogalacturonan component of the cell wall are proposed ligands for WALL-ASSOCIATED KINASES (WAKs) that have epidermal growth factor (EGF)-like domains in their ECD [41]. The EGF-like domain is found exclusively in extracellular proteins or in the extracellular region of transmembrane proteins and functions primarily in mediating protein–protein interactions [42]. OG perception by WAKs has been implicated directly in immunity, and is also speculated to function during symbiotic interactions [43,44]. In addition to pectin-derived molecules, recent parallel studies showed that RKs from the LRR-I family, which have LRR-malectin ECDs, are involved in the perception of cellulose-derived ligands [45–47]. Similar to LRR-XI RKs that perceive peptides, closely related LRR-I RKs have different perception specificities but the biochemical basis for such specificity remains to be determined [45–47].

Lipids from oomycete and bacterial pathogens also elicit immune responses and are perceived by G-type lectin PRRs (also known as S-domain RKs; SD-RKs) [48,49]. The SD-RK LIPOOLIGOSACCHARIDE-SPECIFIC REDUCED ELICITATION (LORE) binds bacterial medium-chain 3-hydroxy fatty acids (3-OH-FA) with a preference for C10:0 FAs [48]. Similarly, RESISTANT TO DFPM-INHIBITION OF ABSCISIC ACID SIGNALING 2 (RDA2) perceives the microbe-specific ceramide D to activate immune signaling [49]. Additionally, two L-type lectin RKs, LecRK-I.1 and LecRK-I.8, are involved in sensing phosphatidylcholine derived from insect eggs [50,51]. L-type lectin RKs have also been implicated in sensing extracellular nucleotides as DAMPs. DOES NOT RESPOND TO NUCLEOTIDES 1 (DORN1, also known as LecRK-I.9) and the related LecRK-I.5 detect extracellular ATP, whereas LecRK-I.8 is a receptor for extracellular NAD^+^ [52–54]. The observation that LecRK-I.8 participates in the perception of both phosphatidylcholine and extracellular NAD^+^ is somewhat surprising and might indicate that additional components contribute to the perception specificity within the L-type lectin RK family.

Similar to animals, the coordinated release of autocrine- and paracrine-acting signaling peptides has emerged as a central concept in the regulation of plant development and environmental response [55]. Many peptide ligands have been structurally characterized in complex with their LRR-RK receptors and several common themes have emerged [56]. However, many novel ligand–receptor pairs await structural characterization. For example, the SERINE RICH ENDOGENOUS PEPTIDES (SCOOPs) that are perceived by MALE DISCOVERER 1-INTERACTING RECEPTOR-LIKE KINASE 2 (MIK2) contain a Ser-X-Ser motif near the center of the bioactive peptide, distinct from other known peptide families [56–59]. Similarly, CTNIP/SCREW (for SMALL PHYTOCYTOKINES REGULATING DEFENSE AND WATER LOSS) peptides, perceived by HAESA-LIKE 3 (HSL3), carry two conserved Cys residues required for function [60,61]. It will be interesting to understand how such novel peptide configurations contribute to ligand recognition. As is the case with peptide recognition by the plant immune system, most endogenously produced ligands are peptides that are perceived by LRR-RKs, however not all LRR-RKs perceive peptides. One notable exception is the BR receptor, BRASSINOSTEROID INSENSITIVE 1 (BRI1) that perceives its steroid ligand via an island domain its ECD [62]. Conversely, some peptides are perceived by other RK families. Peptides from the RAPID ALKALINIZATION FACTOR (RALF) family are perceived by *Catharanthus roseus* RECEPTOR LIKE KINASE 1-LIKE (CrRLK1L)-RKs that have tandem malectin-like domain ECDs [63]. The founding member of the Arabidopsis CrRLK1L family, FERONIA (FER), participates in the perception of most of the 37 RALFs produced in Arabidopsis while other CrRLK1Ls likely sense a narrower set of RALF peptides [64]. CrRLK1L-RKs cooperate in RALF perception with additional components including LORELEI-LIKE glycosylphosphatidylinositol (GPI)-anchored protein 1 (LLG1) and several LRR-extensin (LRX) proteins, although it is unclear whether all the components form a single complex, or whether they perceive RALFs separately [65–67]. FER is also implicated in the perception of POLLEN COAT PROTEIN B peptides during the compatible pollen response in Arabidopsis coordinately with another CrRLK1L, ANJEA as well as LLG1 [68]. Whether LRXs also play a role in this process is unknown.

Although great effort has been expended to understand ligand–receptor relationships across the RK family, the specific ligands for many RKs await discovery. Additionally, many receptor–ligand pairs await biochemical and biophysical characterization to achieve an understanding of the range of ligand-perceiving mechanisms across the RK family.

### Regulation of RK signaling by complex formation

Biochemical analysis indicates ligand-induced receptor/co-receptor dimerization as a common activation mechanism across the RK family. The SOMATIC EMBRYOGENESIS RECEPTOR KINASEs (SERKs), short-ECD LRR-RKs from subfamily LRR-II, function as co-receptors for several large-ECD LRR-RKs in different physiological contexts [69]. The archetypal co-receptor is the subfamily II LRR-RK BRI1-ASSOCIATED RECEPTOR KINASE 1/SERK3 (hereafter, BAK1) [69,70]. BAK1 is characterized by a short ectodomain containing five LRRs, a proline-rich extracellular juxtamembrane domain, an active Arg-Asp (RD)-type cytoplasmic ePK domain, and a C-terminal tail containing several important regulatory phosphorylation sites [71]. Genetic analysis positioned BAK1 as a positive regulator of both BR signaling and PAMP-triggered immunity [72–75]. BAK1 interacts with both BRI1 and FLS2 *in vivo* in a ligand-dependent manner [72–75]. In response to flg22, BAK1 rapidly forms a complex with FLS2, suggesting an important role in the activation of flg22 responses [75]. Given this rapid, ligand-induced association, and the observation that *BAK1* mutant plants did not show reduced flg22 binding, BAK1 was proposed to serve as a co-receptor in the FLS2 complex [74,75].

**Figure 2.**
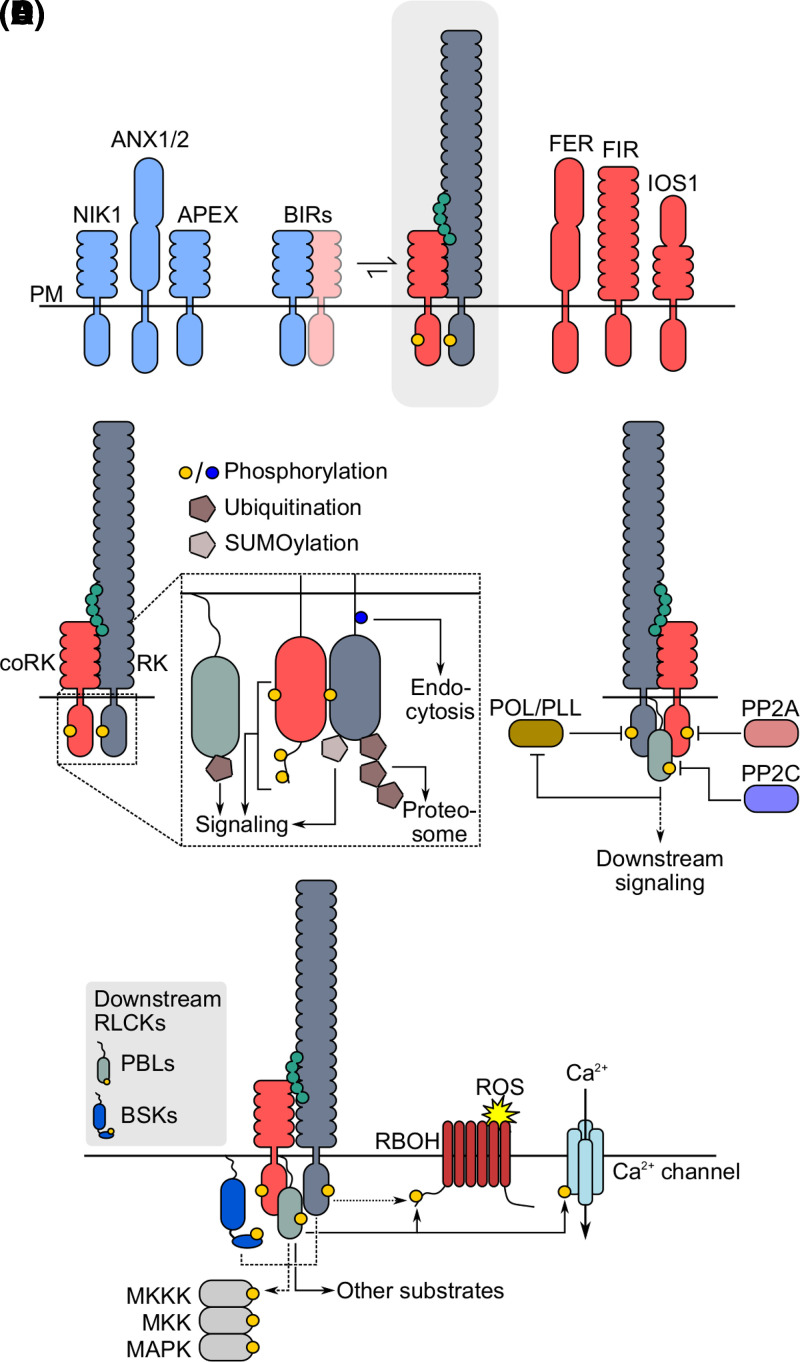
Activation and regulation of RK-mediated signaling. (**A**) Receptor complex formation is triggered by ligand binding (grey box) and is positively (red) and negatively (blue) regulated by several accessory RKs through different mechanisms. Regulatory RKs shown function in PAMP-triggered immune signaling. (**B**) RK cytoplasmic domains are post-translationally modified following ligand perception. Cytoplasmic domain phosphorylation is essential to activate downstream signaling (yellow) and controls RK endocytosis (blue). Poly-ubiquitination leads to RK degradation. Mono-ubiquitination of BIK1 activates immune signaling. SUMOylation of multiple RKs positively regulates downstream responses. (**C**) Regulation of LRR-RK mediated signal activation by protein phosphatases. (**D**) Downstream signaling is primarily executed by RLCKs from the PBL and BSK families. PBLs directly activate RBOHD and Ca^2+^ channels (solid arrows). RBOH are directly activated by ligand-binding receptors in some contexts (dotted arrow). MAPK cascades are activated by RKs, PBLs, or BSKs depending on the receptor complex (dotted arrows). LRR-RKs are shown as representative RKs in **B** and **D**. See text for details.

Structural characterization of the isolated ECDs of FLS2 and BRI1 in complex with their respective ligands and with BAK1 or SERK1 revealed a conserved mechanism of co-receptor recruitment despite dramatically different ligand-binding modes. Recruitment of BAK1 to ligand-bound FLS2 is achieved not only by direct contacts between FLS2 and BAK1, but also contacts between the C-terminus of the ligand and the BAK1 ECD [76]. Similar to FLS2/BAK1/flg22, BAK1 and SERK1 make direct contacts with both BRI1 and the BRI1-bound BR ligand [77,78]. For both receptor complexes, neither ligand binding nor co-receptor recruitment triggers a conformational change in FLS2, BRI1 or the co-receptor [76–78]. Rather, ligand perception creates a new binding surface for BAK1 recruitment. In this context, both flg22 and BR serve as a molecular glue to stabilize the interaction between the receptor and co-receptor. Thus, two LRR-RK receptors that control distinct signaling pathways both activate downstream signaling by recruitment of BAK1 as co-receptor. The characterization of BAK1 in flg22 and BR perception provides the foundational work for the concept of RK activation by co-receptor recruitment across the RK family (Figure 2A). One important exception to the receptor/co-receptor model is the recently identified receptor for PSY (PLANT PEPTIDE CONTAINING SULFATED TYROSINE) peptides. Here, the PSY ligands are proposed to inhibit their receptors, and depletion of the ligand under stress then activates receptor-mediated signaling [79]. Such an activation mechanism is however distinct from phylogenetically related RKs, and thus awaits further biochemical testing.

While LRR-RKs from the SERK family serve as co-receptors in a multitude of signaling pathways, not all known LRR-type ligand-binding receptors are activated by SERK co-recruitment [69]. This observation prompted the search for additional short-ECD LRR-RKs that might fulfill co-receptor functions, most notably in meristem maintenance and vasculature differentiation in the root regulated by CLAVATA1 (CLV1) and the related BARELY ANY MERISTEM (BAM) 1, 2, and 3 LRR-RKs [80,81]. CLV1 and BAMs perceive peptides from the CLAVATA3/EMBRYO SURROUNDING REGION (CLE) family to regulate different aspects of plant development [82]. The LRR-II clade, which includes the SERKs, consists of 14 members in Arabidopsis, and parallel genetic studies converged on additional members as co-receptors in CLE perception [80,81,83]. The LRR-RK CLE-RESISTANT RECEPTOR KINASE (CLERK, also known as CLAVATA3 INSENSITIVE RECEPTOR KINASE 2, CIK2) was identified in a combined transcriptomics/forward genetics approach as a regulator of CLE-dependent protophloem differentiation in Arabidopsis [80]. *CLERK* transcript is down-regulated by CLE26 perception and loss-of-function *clerk* mutants are insensitive to root growth inhibition triggered by several CLE peptides. Similarly, CLERK/CIK2 and three additional CIKs (CIK1, 3, and 4) are important for regulation of the shoot apical meristem as indicated by an enlarged meristem in *cik1,2,3,4* quadruple mutants reminiscent of *clv1* mutants [83]. CIKs have additional functions in anther development, a process involving BAM1 and BAM2 [81]. CLERK/CIK2 associates with RKs that control meristem maintenance and is phosphorylated in response to CLAVATA3 (CLV3), the CLV1 ligand [83]. Despite the clear genetic implication of CLERK/CIKs in meristem regulation, biochemical evidence to support their function as *bona fide* co-receptors for CLV1 or the BAMs is lacking, and attempts to resolve a SERK-like co-receptor mechanism *in vitro* have so far been unsuccessful [80]. Whether the binding affinities are too weak for *in vitro* studies or whether additional unknown molecular components are required for CLERK/CIK co-receptor function, remains an open question.

LysM-RK complexes involved in polysaccharide perception also activate downstream signaling through co-receptor recruitment. Intriguingly, a common co-receptor — CHITIN ELICITOR RECEPTOR KINASE 1 (CERK1) — participates in both symbiotic and immune signaling through distinct LysM receptor complexes [16,17,84–87]. Two LysM-RKs, LYSM RECEPTOR KINASE 4 (LYK4) and LYK5, perceive chitin oligomers to activate immune signaling [14,15,87]. Upon chitin perception, CERK1 associates with LYK5 to activate signaling [15]. LYK4 can additionally associate with CERK1 in a chitin-dependent manner; although this association requires LYK5 [88]. Both LYK4 and LYK5 have negligible protein kinase activity *in vitro* [14,15], suggesting that the active kinase CERK1 is responsible for initiation of signaling after recruitment to LYK4/LYK5. LYK4 and LYK5 additionally function in localized chitin-triggered signaling at plasmodesmata with LYM2 [89]. Thus, ligand-induced co-receptor recruitment by immune-associated LysM-RKs mirrors the activation mechanism described for LRR-RK-mediated signaling. In the model legume *Lotus japonicus*, LjCERK6 (MtCERK1 in *Medicago truncatula*), participates in chitin-induced immune signaling [90]. Chitin-induced immune signaling in rice is also activated by recruitment of OsCERK1 to a high-affinity chitin receptor, although in this context chitin is perceived by the LysM-RP CHITIN ELICITOR BINDING PROTEIN (CEBiP) rather than a LysM-RK as in Arabidopsis [91].

LysM-RKs also play critical roles as initiators of signal transduction in both root nodule and mycorrhizal symbioses. In *Lotus*, NOD FACTOR RECEPTOR 1 (NFR1) and NFR5 (LYK3 and NFP, respectively in *Medicago*) are the receptors for Nod factors derived from rhizobial symbionts that trigger the nodulation process [16,17]. Genetic experiments indicate that both receptors are required for the cellular response to Nod factors, suggesting that NFR1 and NFR5 might function together in Nod factor-induced signaling [16,17]. NFR1 and NFR5 directly interact, and cell death induced by NFR5 overexpression in *N. benthamiana* requires co-expression of functional NFR1, collectively indicating that NFR1 and NFR5 form an active receptor/co-receptor complex [92]. Consistent with a receptor/co-receptor model, a recent synthetic biology approach demonstrated that the association of NFR1 and NFR5 in roots is sufficient to initiate root nodule formation [93]. Interestingly, a second LysM-RK, NFRe (for epidermal NOD FACTOR RECEPTOR), supports responses to rhizobial-derived Nod factors mediated by NFR1 and NFR5 [94]. Like NFR1, NFRe directly binds Nod factors, associates with, and transphosphorylates NFR5 [94,95]. Genetic analysis of *NFR1* and *NFRe* indicates that NFRe regulates a spatially restricted set of Nod factor responses [94], suggesting that NFR5 controls distinct spatio-temporal aspects of the nodulation process through different co-receptors, namely NFR1 and NFRe. OsCERK1, the rice ortholog of NFR1, participates in both mycorrhizal symbiosis and immunity [84,85]. Surprisingly, neither CEBiP or the rice ortholog of NFR5 (OsNFR5) function in the perception of Myc factors from mycorrhizal fungi [85], suggesting that OsCERK1 functions as a co-receptor in distinct receptor complexes in symbiosis and immunity. Recently, an additional LysM-RK with a role in Myc factor perception was identified in rice. OsLYK2 is genetically required for Myc factor responses in rice roots and can form a ligand-induced complex with OsCERK1 [96]. Collectively, the characterization of NFR1/CERK1 across plant species with the capacity for microbial symbioses reveals a function not dissimilar to that of BAK1 in LRR-RK complexes, where CERK1 functions as a co-receptor for a repertoire of different LysM-containing receptors (either RKs or RPs) to regulate distinct physiological processes.

By comparison to the heterocomplexes observed for LRR- and LysM-RKs, SD-RKs are proposed to undergo homodimerization important for ligand-induced signaling [97–102]. The SD-RK SRK (for S-locus Receptor Kinase) is involved in the self-incompatibility response in the Brassicaceae, together with its cognate ligand, S-locus cysteine-rich protein (SCR). *In vitro*, isolated SRK ectodomains homodimerize in a ligand-dependent manner involving contact between both receptor monomers and the receptor ECDs and SCR [98,99]. Cross-linking experiments indicate that SRK is self-associated *in vivo*, suggesting that the native membrane environment might influence the multimeric state of SRK [100]. Further insight into the functional organization of SD-RKs comes from characterization of the PRR LORE. Like SRK, LORE self-associates *in vivo* in a manner that is influenced by both its transmembrane domain and ECD [97]. Homodimerization is not required for ligand perception by LORE but is necessary for 3-OH-C10:0-induced immune signaling [48,97,103]. Given the apparent multimeric state of SD-RKs, it is not clear how ligand binding to a preformed complex would activate signaling. Whether SD-RKs are activated by conformational rearrangements or whether yet unknown co-receptors participate in the activation process remains an open question.

The recruitment of co-receptors by ligand-bound RKs is a key step in the activation of downstream signaling for several RK families. It is thus not surprising that co-receptor recruitment is a tightly regulated process, necessary to limit the activation of intracellular signaling in the absence of a specific ligand. Much of our understanding of the regulation of receptor complex formation comes from the characterization of immune signaling mediated by FLS2 and EFR in Arabidopsis. Here, multiple ‘accessory’ RKs control ligand-induced complex formation either directly by interaction with one or both receptor components, or indirectly by as yet unknown mechanisms (Figure 2A). The most well-characterized regulators of receptor complex formation in immune signaling are the BAK1-INTERACTING RECEPTOR KINASES (BIRs). The BIRs make up a four-member subgroup of the LRR-X family, and two members — BIR2 and BIR3 — were initially identified as interactors of BAK1 by immunoprecipitation mass spectrometry [104]. Like BAK1, the BIRs have short ECDs but, distinctly, have intracellular pseudokinase domains rather than active ePK domains. Genetic analysis of *BIR2* and *BIR3* demonstrates that both function as negative regulators of immune signaling, with BIR3 functioning additionally in the BR signaling pathway via interaction with BRI1 [104,105]. *In vivo* biochemical experiments indicate that BAK1 is released from BIR2 upon stimulation with immune eliciting ligands [104]. Structural analysis of the BAK1 or SERK1 ECD in complex with BIR1 or BIR3, respectively, shows that the BIRs occupy an interaction interface on BAK1/SERKs that is required for engagement of the ligand-bound receptor [106,107]. Receptor complex activation occurs because the ligand-bound receptor can outcompete BIRs for BAK1 binding. Sequestration of BAK1 by BIRs thus prevents receptor complex activation in the absence of appropriate stimuli.

Two additional short ectodomain RKs — namely, NUCLEAR SHUTTLE PROTEIN-INTERACTING RECEPTOR KINASE 1 (NIK1) and APEX — negatively regulate FLS2/BAK1 complex formation, but likely through different mechanisms. NIK1 can associate with both BAK1 and FLS2 in a flg22-dependent manner and this association requires FLS2. Loss-of-function *nik1* mutants have enhanced elicitor responses, indicating negative regulation of FLS2-mediated immune signaling. Consistent with enhanced elicitor responses, *nik1* mutants also show increased elicitor-induced FLS2/BAK1 complex formation [108], suggesting that NIK1 negatively regulates immune signaling by directly disrupting receptor/co-receptor interactions in the active receptor complex. Similar to *NIK1*, loss-of-function mutants of *APEX* show increased elicitor-induced FLS2/BAK1 complex formation and enhanced immune responses [109]. APEX interacts weakly with FLS2 *in vitro*, but it is not clear whether this occurs *in vivo* [109]. As such, the mechanistic basis for regulation of FLS2/BAK1 complex formation by APEX is unknown. Interestingly, both NIK1 and APEX are members of the LRR-II subfamily that contains the SERKs, and both have overall architectures similar to the SERK proteins. It will therefore be interesting to understand whether NIK1 and APEX themselves serve as co-receptors for other LRR-RKs and how this might relate to their roles in immune regulation. Indeed, NIK1 is closely related to the CIK co-receptors that function in meristem maintenance, suggesting a potential co-receptor function.

In the same study that identified APEX, an additional LRR-RK, FLS2-INTERACTING RECEPTOR (FIR), was identified as an interactor of FLS2 [109]. In contrast with APEX, *fir* mutants show a substantial impairment of ligand-induced FLS2/BAK1 complex formation and an attenuation of downstream signaling, indicating that FIR is a positive regulator of elicitor-induced immune responses by supporting receptor complex activation. Like APEX, it is not known whether FLS2/BAK1 and FIR interact *in vivo*. Thus, further work is required to resolve fully the mechanisms through which FIR regulates the immune response.

Immune signal activation through FLS2/BAK1 complex formation is also regulated by a suite of RKs with malectin-like domain containing ECDs. Among these, the malectin-LRR-RK IMPAIRED OOMYCETE SUSCEPTIBILITY 1 (IOS1) positively regulates immune responses upon perception of flg22 or elf18. Consistently, *ios1* mutants have increased susceptibility to the biotrophic pathogen *Pseudomonas syringae*. IOS1 associates constitutively with FLS2 and BAK1, and positively regulates ligand-induced FLS2/BAK1 complex formation [110]. Surprisingly, loss of IOS1 function does not impair elicitor-induced phosphorylation of the downstream RLCK BOTRYTIS INDUCED KINASE 1 (BIK1) even though BIK1-dependent processes are disrupted in *ios1* mutants.

Three members of the CrRLK1L family — FER, and the closely related ANXUR1 and ANXUR2 (ANX1 and ANX2, respectively) — differentially regulate FLS2/BAK1 complex formation and subsequent flg22 responses. FER functions as a positive regulator in plant immunity as a putative scaffold protein supporting the formation of an active FLS2/BAK1 complex [111]. Such protein scaffold functions are not strictly defined and the specific mechanism of FER function in this context is not currently known. Notably, this function does not require the catalytic activity of FER, as a catalytically deficient mutant of FER can complement impaired FLS2/BAK1 complex formation in the *fer-4* mutant [112]. Conversely, the scaffold function of FER is negatively regulated by its ligand RALF23, in a process that requires its catalytic function, and which controls the nanoscale organization of FLS2 and BAK1 in the PM [112]. By comparison, the CrRLK1Ls, ANX1 and ANX2 negatively regulate FLS2/BAK1-mediated immune signaling. *anx1* and *anx2* mutants have elevated responses to immune elicitors and ANX1 directly associates with FLS2 and BAK1. ANX1 overexpression inhibits FLS2/BAK1 complex formation, suggesting that ANX1 inhibits immune responses through direct action on the receptor complex [113]. Whether the function of ANX1 and ANX2 in PRR complex regulation is controlled by RALF peptide perception is unknown.

Some LRR-RKs, particularly those involved in development, are controlled by proteins from the MEMBRANE ASSOCIATED KINASE REGULATOR (MAKR) family. MAKRs are intrinsically disordered but have linear motifs involved in membrane association, 14-3-3 binding, and in interaction with RK cytoplasmic domains [114]. BRI1 KINASE INHIBITOR 1 (BKI1), the founding member of the MAKR family in Arabidopsis, negatively regulates BR signaling [115]. The C-terminus of BKI1 interacts directly with the BRI1 protein kinase domain and limits association of the BRI1 and BAK1 cytoplasmic domains. Upon ligand perception, BRI1 phosphorylates the membrane-binding region of BKI1, resulting in its dissociation from the membrane, permitting interaction between the BRI1 and BAK1 cytoplasmic domains and consequent activation of the receptor complex [116]. Similarly, MAKR2 genetically negatively regulates TRANSMEMBRANE KINASE 1 (TMK1)-mediated rapid auxin responses. MAKR2 directly interacts with and is phosphorylated by TMK1, indicating a regulatory mechanism similar to that for BRI1 and BKI1 [117]. In contrast, MAKR5 genetically positively regulates BAM3-mediated CLE45-induced signaling in root protophloem development. However, MAKR5 does not directly interact with BAM3, suggesting a different mode of action for MAKR5 compared with BKI1 and MAKR2 [118]. It is noteworthy that, so far, MAKR proteins are only known to function in pathways controlling developmental processes. Whether MAKRs function in other LRR-RK-mediated signaling pathways and whether MAKRs regulate non-LRR-type RKs remain open questions.

### RK signaling is regulated by post-translational modifications

Immediately after ligand-induced formation of the RK complex, phosphorylation of the intracellular domains occurs. The intracellular domains of RKs consist of a protein kinase core flanked by juxtamembrane and C-terminal tail regions — all of which can be the target for different regulatory post-translational modifications. Phosphorylation on RK complexes is widespread, including on Tyr, which has recently emerged as a critical post-translational modification (PTM) regulating early activation events in RK complexes. For a detailed discussion of Tyr phosphorylation in plants, we refer readers to our recent review [119]. Early work on BRI1 and BAK1 indicated that reciprocal transphosphorylation was the primary biochemical event leading to activation the BR receptor complex, although such reciprocal phosphorylation events are not a requirement in other RK complexes [120–122]. The major target sites for these early phosphorylation events are residues within the activation loop — a hallmark for activation across the ePK family [123]. Phosphorylation of the activation loop of both BRI1 and BAK1 is required for BR signaling, and similarly is required for BAK1 function in immune signaling [120,122,124]. Interestingly, EFR — despite its identity as a non-RD protein kinase — also requires the phosphorylation of two sites in its activation loop to trigger responses to its cognate ligand [121]. RKs additionally undergo phosphorylation outside their core protein kinase domains (Figure 2B), and although the phosphorylation of some such sites has demonstrated physiological importance, the mechanistic basis for regulation of RK function by most known phosphorylation events is unclear. Phosphorylation of the juxtamembrane region of multiple RKs including BRI1 and FLS2 has been demonstrated to control ligand-induced endocytosis of RKs [125–128]. C-terminal tail phosphorylation also regulates RK function. BRI1 is phosphorylated on multiple residues in its C-terminal tail and collectively C-terminal tail phosphorylation positively regulates BRI1-mediated signaling, possibly through the relief of BRI1 inhibition exerted by its C-tail [129]. Recent work indicates the importance of phosphorylation of the C-terminal tail of the common co-receptor BAK1, and interestingly, phosphorylation of the BAK1 C-terminal tail is required for only a subset of BAK1 functions [71]. Interestingly, other RKs in the LRR-II subclade, such as CLERK/CIKs and APEX, have conserved sequences at their extreme C-termini that includes the regulatory Ser612 phosphorylation site of BAK1, suggesting a conserved mechanism of co-receptor activation [71,80,81].

Given that RK complexes are activated by phosphorylation, it comes as no surprise that the inactive state of RKs is maintained by a suite of protein phosphatases. Protein phosphatases from the PP2A and PP2C families directly act on components of the receptor complex, and an additional PPC2 targets the downstream RLCK BIK1 for dephosphorylation (Figure 2C) [130–133]. Recent comparative analysis of RK function in immunity and in CLE peptide signaling revealed a conserved phosphatase module regulating the activation state of RKs (Figure 2C) [131]. PP2Cs from the POLTERGEIST (POL) subfamily directly associate with ligand-binding RKs — POL-LIKE 4 (PLL4) associates with both FLS2 and EFR, and directly dephosphorylates the phosphorylated EFR cytoplasmic domain *in vitro*. Genetically, PLL4 and the related PLL5 negatively regulate responses to the bacterial elicitors elf18 and flg22, and the DAMP, AtPep1, indicating that these two phosphatases function broadly to suppress immune activation [131]. Similarly, POL — which was originally genetically implicated in meristem regulation — associates with the CLE peptide receptors CLV1 and BAM3, and functions as a negative regulator of CLE-dependent responses downstream of BAM3. In both contexts, the downstream RLCKs BIK1 and PBL34 phosphorylate N-terminal regions of PLL4/5 and POL/PLL1, respectively, to release the phosphatases from the receptor complex [131,134]. Such a conserved regulatory module functions as a brake on receptor complex activation, and is likely to operate in other RK-mediated pathways as well.

In addition to phosphorylation, RK ubiquitination plays a key role in regulating RK-mediated signaling (Figure 2B). Like phosphorylation, ubiquitination is a reversible PTM, but can uniquely occur in many forms including poly-, mono-, or multi-mono-ubiquitination, with distinct forms differentially affecting protein function [135]. The functional relevance of ubiquitination for RK function was realized when it was discovered that the bacterial effector AvrPtoB from *Pseudomonas syringae* could poly-ubiquitinate the immune receptor FLS2 to promote its degradation [136]. Importantly, the targeting of FLS2 for poly-ubiquitination enhanced virulence of pathogen. At the same time, it was observed that treatment with the FLS2 ligand, flg22, could on its own trigger ubiquitination of the receptor, suggesting that endogenous ligases might also regulate FLS2 function. Indeed, two E3 ubiquitin ligases from the PLANT U-BOX (PUB) family, PUB12 and PUB13, were identified as endogenous regulators of FLS2 targeting the receptor for poly-ubiquitination and subsequent degradation. Interestingly, PUB12 and PUB13 are substrates of the co-receptor BAK1, indicating that the receptor complex directly activates its own turnover [137]. PUB12 and PUB13 target additional receptors for degradation including components of the chitin receptor complex (LYK5 and CERK1) as well as BRI1 [128,138,139]. Turnover of ERECTA (ER), a LRR-RK regulating developmental patterning, is similarly regulated by PUB30 and PUB31 [140]. The regulation of RK-mediated signaling by PUB E3 ubiquitin ligases is reminiscent of the POL/PLL phosphatase module repressing complex activation, and likely represents a mechanism regulating many RK complexes. Consistently, proteomic analysis identifies many RKs and RLCKs as targets of ubiquitination [141,142]. In addition to ubiquitination, both BRI1 and FLS2 are modified on Lys by the SMALL UBIQUITIN-LIKE MODIFIER (SUMO). In both contexts, SUMOylation positively regulates receptor function [143,144].

Downstream of RK complexes, RLCKs are also regulated by ubiquitination, as is exemplified by the immune RLCK, BIK1. BIK1 is a target for multiple ubiquitin ligases and is subject to both poly-ubiquitination and mono-ubiquitination [145,146]. Two ubiquitin ligases from the PUB family, PUB25 and PUB26, target BIK1 for poly-ubiquitination and subsequent proteasome-mediated degradation. PUB25 and PUB26 can target BIK1 under both steady-state and ligand-stimulated conditions; however, the pool of BIK1 activated by the receptor complex is resistant to such targeting, permitting activation of downstream signaling. Consistent with the targeting of BIK1 for degradation, *pub25 pub26* mutants show enhanced responses to immune elicitors [145]. BIK1 is additionally a target for mono-ubiquitination by a different set of ubiquitin ligases — RING-H2-FINGER A 3A (RHA3A) and the related, RHA3B. Here, RHA3A and RHA3B mono-ubiquitinate BIK1 following ligand perception by the receptor complex, triggering the dissociation of BIK1 from activated FLS2/BAK1. Interestingly, nine different sites on BIK1 are ubiquitinated by RHA3A/RHA3B, and all nine must be mutated to abolish BIK1 mono-ubiquitination. Mono-ubiquitination of BIK1 is suggested to be required for the activation of immune signaling as a non-modifiable variant could not fully activate elicitor-induced immune responses *in planta* [146]. It remains an open question, however, how mono-ubiquitination in a seemingly non-site-specific manner could equivalently regulate BIK1 function. Nevertheless, it seems likely that other RLCKs will be similarly regulated by both poly- and mono-ubiquitination to control RK-mediated signaling.

### RKs activate RLCKs as executors of downstream signaling

The majority of characterized RKs trigger a common set of rapid signaling processes including the flux of Ca^2+^ into the cytosol, the production of reactive oxygen species (ROS) in the apoplast by PM-localized NADPH oxidases, and the initiation of cytosolic mitogen-activated protein kinase (MAPK) cascades (Figure 2C) [147–149]. Receptor complex activation is linked to downstream signaling events by RLCKs. RLCKs are phylogenetically related to the RKs except that they lack extracellular ligand-sensing domains [4,150]. In Arabidopsis, approximately one quarter of the RKs (∼150 genes) are classified as RLCKs. Most RLCKs are tethered to the PM either via a transmembrane helix, or more commonly through myristoylation and palmitoylation at their N-termini [150]. Among RLCKs, two subfamilies — the RLCK-VII clade, AVRPHB SUSCEPTIBLE 1 (PBS1)-LIKEs (PBLs) and the RLCK-XII clade, BRASSINOSTEROID SIGNALING KINASEs (BSKs) — have received a great deal of attention for their critical roles in both development and immunity. Owing to the wealth of information regarding the function of PBLs and BSKs, we focus here on these two groups, but do not discount the important functions of RLCKs from other clades.

The first RLCK documented to function downstream of an RK in plants was the M locus protein kinase (MLPK) that functions as a positive regulator of the self-incompatibility response in *Brassica* species [151]. MLPK directly associates with SRK and requires PM localization for its function, indicating a role as the primary executor of signaling downstream of SRK [152]. MLPK belongs to the RLCK-VII family and is orthologous to PBL9 and PBL10 from Arabidopsis; although little is known about the function of these two proteins. The RLCK-VII family consists of 46 members in Arabidopsis and several members are known to function in plant development or immunity [150,153]. Indeed, the RLCK-VII proteins BIK1 and its paralog PBL1 are central positive regulators of immune signaling, acting directly downstream of several immune RKs [58,59,154–156]. Interestingly, BIK1 functions as a negative regulator of immune signaling downstream of the LRR-RLP, RLP23 [157], but the mechanisms underlying the opposing roles of BIK1 in LRR-RK versus LRR-RLP mediated immunity are unclear. Instead, two other PBLs, PBL30 and PBL31, positively regulate immune signaling downstream of RLP23 [158]. Additional PBLs function as positive regulators downstream of CERK1 (PBL27 and RLCK-VII-4) and LORE (PBL34, 35, and 36) in chitin- and 3-OH-C10:0-induced immunity, respectively [153,159–161]. Beyond immunity, several PBLs control signaling downstream of RKs involved in growth and development. The PBL, CONSTITUTIVE DIFFERENTIAL GROWTH 1 (CDG1) regulates BR signaling downstream of BRI1 through phosphorylation of the phosphatase, BRI1 SUPPRESSOR 1 (BSU1) [162], and is additionally reported to function in immunity through controlling the abundance of immune receptors [163]. Intriguingly, the three PBLs that initiate immune signaling downstream of LORE also function as mediators of CLE peptide signaling downstream of CLV1 and the BAMs [131], raising the question of how specificity is derived in these two distinct signaling pathways. Finally, the RLCK SGN1 (PBL15) regulates the localized production of ROS required for deposition of the Casparian strip in developing roots [148]. Thus, PBLs as executors of signaling represents a common theme in RK signaling.

A second family of membrane-associated RLCKs — the BSKs — also function downstream of multiple RKs and in different physiological contexts. BSKs associate with the PM via N-terminal lipid modifications, and are reported to be active protein kinases [164]. BSKs additionally carry a regulatory TETRATRICOPEPTIDE REPEAT (TPR) domain C-terminal to their protein kinase domain that likely functions to inhibit the protein kinase [165]. BSKs were identified in proteomics studies as PM proteins up-regulated in response to BR treatment and are substrates of the BR receptor complex [166]. Genetic characterization of several BSKs indicated a role in BR signaling, and placed them upstream of the BRASSINOSTEROID-INSENSITIVE 2 (BIN2) GSK3 kinase in the signaling pathway [166]. In addition to BRI1, BSKs were later found to associate with multiple RKs, in particular with RKs regulating immunity [167,168]. Accumulated genetic evidence implicates BSKs in the regulation of multiple signaling branches downstream of immune RK complexes [164,167,169–171]. Three BSKs, BSK1, BSK2, and BSK12 (also called SHORT SUSPENSSOR, SSP), function downstream of the LRR-RK ERECTA in the establishment of zygote polarity [165,172]. As is the case for some PBL family members, the association of BSKs with multiple RKs in distinct physiological contexts raises the question of how specificity is derived downstream of RK activation.

The first substrate known to be phosphorylated by a RLCK downstream of receptor complex activation was the NADPH oxidase RESPIRATORY BURST OXIDASE HOMOLOG D (RBOHD) that is responsible for generation of the apoplastic oxidative burst during immune signaling. BIK1/PBL1 phosphorylate multiple residues in the N-terminal cytosolic tail of RBOHD that are required for its activation [173,174]. Such a signaling module is evolutionarily ancient in the plant lineage, as indicated by the conservation of PBL-mediated activation of a RBOH during chitin perception in *Marchantia polymorpha* [175]. Another member of the PBL family — PBL13 — negatively regulates both RBOHD and its accumulation through phosphorylation at its C-terminus [176]. Interestingly, other members of the PBL family regulate RBOH proteins downstream of receptor complexes in non-immune contexts. In the root, CIF peptides signal through an LRR-RK known as SCHENGEN 3 (SGN3) to control formation of the Casparian strip — an apoplastic barrier in the root endodermis [177]. Downstream of SGN3, SGN1 (PBL15) phosphorylates and activates RBOHD and RBOHF to promote Casparian strip formation [148]. The RKs HAESA (HAE) and HAE-LIKE 2 (HSL2) control cell separation during floral organ abscission. This process is regulated by the INFLORESCENCE DEFICIENT IN ABSCISSION (IDA) peptide that is a ligand for HAE and HSL2. Like other RK-dependent pathways, IDA perception by HAE/HSL2 triggers ROS production and Ca^2+^ fluxes in a receptor-dependent manner [149]; although the RLCKs, NADPH oxidases, and Ca^2+^ channels functioning in this context are unknown. Notably, no RLCK is currently known to link ATP perception with NADPH oxidase activation. Instead, the ATP receptor, DORN1, is proposed to directly phosphorylate and activate RBOHD following ligand perception [52].

Early studies using Ca^2+^ reporter lines implicated cytosolic Ca^2+^ fluxes in RK-dependent signaling, and BIK1 and PBL1 were reported to act upstream of Ca^2+^ signaling during immunity, likely through the regulation of multiple independent Ca^2+^ channels [178–181]. The guard cell-expressed, Ca^2+^-permeable channels OSCA1.3 and OSCA1.7 function coordinately in elicitor-induced stomatal closure and BIK1-dependent, site-specific phosphorylation of OSCA1.3 is required for this function [182]. Similarly, CYCLIC NUCLEOTIDE GATED CHANNEL 4 (CNGC4) that contributes to elicitor-triggered Ca^2+^ fluxes under specific Ca^2+^ concentrations is a substrate of BIK1 [183]. A Ca^2+^ permeable channel formed by CNGC2 and CNGC4 is inhibited by calmodulin (CaM) and BIK1-dependent phosphorylation of CNGC4 on at least three sites reduces the sensitivity of the channel to CaM [183], permitting Ca^2+^ flux through the channel during immune signaling under specific external Ca^2+^ concentrations. Collectively, the balance of evidence suggests that RK activation converges on PBL-controlled apoplastic ROS production and Ca^2+^ fluxes in multiple physiological contexts.

In addition to Ca^2+^ fluxes and apoplastic ROS production, MAPK cascades fulfill important signaling functions downstream of RK activation. The majority of known MAPK substrates are transcription factors, suggesting that the activation of MAPK cascades might be important for altering transcriptional landscapes in different physiological contexts [184]. RLCKs from both the PBL and BSK families have been linked to MAPK cascade activation downstream of RK complexes. In other cases, ligand-binding receptors might directly activate MAPK cascades via interaction with and phosphorylation of MAP3Ks [185]. In immunity, PBL27 as well as PBLs from the RLCK-VII-4 subclade are required for MAPK signaling downstream of chitin perception by CERK1 [153,159,161,186]. It is not clear whether PBLs activate MAPK signaling downstream of other immune receptors, however such a role might be fulfilled by BSKs [153,159,164,186,187]. MAPK cascades also operate in developmental contexts, for example downstream of ER and HAE/HSL2 [188,189]. While the biochemical links between HAE/HSL2 and the MAPK pathway are unknown, genetic experiments position BSK1 and BSK2 upstream of YODA in ER-mediated signaling. Additionally, YODA-dependent MAPK signaling can be activated in a receptor-independent manner by the non-canonical BSK, SSP [165,172]. Given that BSKs function downstream of a multitude of RKs, it will be interesting to see whether they function upstream of MAPK cascades in other contexts, particularly in BR signaling where BSKs were first discovered.

## Five critical questions in plant receptor kinase signaling

Three decades of intensive study has led to a comprehensive understanding of the physiological processes regulated by RKs and the functionally related RLPs. Indeed, RKs contribute to the entire life cycle of the plant from fertilization to post-embryonic development and seed set. RKs are essential components in the sensing of non-self or damaged-self, both in the context of immune activation and symbiotic relationships. RKs and RLPs fulfill these critical functions through (1) expanded families of receptors having architectures capable of signal perception and transmission across the membrane; (2) a diverse repertoire of ECDs facilitating the perception of an equally diverse repertoire of ligands; (3) the tight regulation of signal activation through a common mechanism of co-receptor recruitment; (4) the coordinated activation and attenuation of signaling regulated by post-translational modifications of different types; and, (5) the activation of common set of downstream signaling processes to drive adaptive changes in cellular physiology. From these five themes emerge several critical questions that remain to be addressed by our research community.

### Plant genomes encode hundreds of RKs and secreted signaling peptides — what are they all doing?

Plants RK and signaling peptide gene families have undergone considerable expansion, in some cases in a lineage-specific manner. A critical task in the next decade will be to disentangle these large gene families with the aim of understanding ligand and receptor function and identifying ligand–receptor pairs. Additionally, whether all encoded RKs have ligands or whether some represent a reservoir for neofunctionalization, remains an open question. In this regard, a combination of genomics and bioinformatics approaches might be informative to comprehensively identify the diversity of signaling peptides encoded by plants, as has been recently achieved for RK families across the green lineage. Gene family co-expansion within plant lineages might inform the identification of peptide-receptor pairs. To understand peptide and receptor function, a classical approach characterizing the tissue- and cell-type specific spatial domains of peptide and receptor expression within the plant, for example with promoter–reporter fusions, will help to guide reverse genetic approaches, especially for large gene families.

### Many RKs implicated in the sensing of microorganisms have no known ligands — what do they perceive?

RKs carry a diverse suite of ECDs suggestive of the capacity to detect a large repertoire of ligands. One hypothesis for the evolutionary acquisition of such RK architectures is that it facilitated the detection of non-self organisms during the colonization of land. What novel non-self ligands might be perceived by the many orphan receptors in plants? Although the characterization of novel peptide ligands can be relatively straightforward, other types of ligands such as lipids and carbohydrates represent unique challenges. Here, close collaborations with chemists specialized in the production of ligands labeled with novel chemistries useful in the isolation of receptors might provide a path forward for comprehensively understanding how plants sense their microbiota.

### Receptor complex formation is tightly regulated — what is the minimal composition of a receptor complex that is required to activate signaling?

Complex formation with a co-receptor is thought to be the minimal activating event for multiple RKs families and this process is tightly regulated by accessory RKs. Can a minimal complex capable of *ligand*-*activated* signaling be defined? In cases where RK complexes might be activated conformationally (e.g. SRKs and LORE), how might rearrangement of ECDs drive activation of cytoplasmic domains? The answers to these questions will come from detailed structural analysis of RKs in complex with their ligands and from the characterization of receptor function in heterologous systems. Although a wealth of structural data on isolated ECDs is available, the key technical leap will be to structurally characterize full-length receptors and receptor complexes. These challenging questions will be best addressed by multi-disciplinary teams of plant and structural biologists, and will be facilitated by advanced imaging technologies such as cryo-electron microscopy.

### RK complexes contain (at least) pairs of protein kinases — what is the biochemical basis for the activation of cytoplasmic protein kinase domains?

For a long time, it was though that dimerization of RK ECDs facilitated reciprocal transphosphorylation via cytoplasmic domain proximity. This simple model has recently been challenged by the suggestion that allosteric processes might also participate in RK cytoplasmic domain activation in a manner analogous to animal RTKs. Biochemical and structural approaches comparing different types of RKs will clarify whether multiple modes of activation are possible, or whether RKs activate through a conserved mechanism. Similarly, a deeper understanding of the role of PTMs — particularly phosphorylation — will be required to understand how ligand perception is translated into downstream signaling events. Here, time-resolved (phospho)proteomic analysis of RK signaling will be informative.

### Many receptor kinases activate common downstream events — how is specificity generated in RK-mediated signaling?

A central theme that has emerged in the last decade is that RKs often activate a common core of downstream signaling processes, in addition to the activation of pathway-specific processes in some contexts. Most characterized RKs activate Ca^2+^ fluxes, apoplastic oxidative bursts, and MAPK phosphorylation cascades, and do so through a shared set of RLCKs as the primary executors of cytosolic signaling. What then makes the activation of one RK distinct from another? This is a long-standing question for our research community, and our view is that sufficient answers have not yet emerged in the literature. Understanding specificity in signal transduction will require a multi-faceted approach with significant contributions from advanced systems and cell biological approaches. Tissue- and cell-type specific expression of RKs likely contributes substantially to their specific functions. Here, single cell RNA-seq will provide important resolution on domains of RK and ligand expression as well as transcriptional landscapes regulated by RKs, working under the hypothesis that specificity might emerge further downstream, possibly in a tissue- and/or cell-type specific manner. Super-resolution microscopy will be an important tool for understanding the spatial separation of RK signaling within the cell. Finally, proteomics approaches such as proximity-labeling mass spectrometry will help to understand the network architecture of plant signal transduction pathways.

We think that that there is urgent need to develop a holistic understanding of RK function. Large secreted signaling peptide and RK gene families render this a formidable, but necessary task, given the likely importance of RKs as key targets for biotechnological applications aimed at stabilizing agricultural outputs in the coming decades.

## Abbreviations

BIK1: BOTRYTIS INDUCED KINASE 1
BIRs: BAK1-INTERACTING RECEPTOR KINASES
BSKs: BRASSINOSTEROID SIGNALING KINASEs
CEBiP: CHITIN ELICITOR BINDING PROTEIN
CERK1: CHITIN ELICITOR RECEPTOR KINASE 1
CLE: CLAVATA3/EMBRYO SURROUNDING REGION
CLV1: CLAVATA1
CNGC4: CYCLIC NUCLEOTIDE GATED CHANNEL 4
CrRLK1L: *Catharanthus roseus* RECEPTOR LIKE KINASE 1-LIKE
DAMPs: damage-associated molecular patterns
ECD: extracellular domain
EFR: ELONGATION FACTOR Tu RECEPTOR
EGF: epidermal growth factor
ER: ERECTA
FER: FERONIA
FIR: FLS2-INTERACTING RECEPTOR
FLS2: FLAGELLIN SENSING 2
HAE: HAESA
HSL2: HAE-LIKE 2
IDA: INFLORESCENCE DEFICIENT IN ABSCISSION
IOS1: IMPAIRED OOMYCETE SUSCEPTIBILITY 1
IRAKs: INTERLEUKIN-1 RECEPTOR ASSOCIATED KINASE
LLG1: LORELEI-LIKE glycosylphosphatidylinositol (GPI)-anchored protein 1
LORE: LIPOOLIGOSACCHARIDE-SPECIFIC REDUCED ELICITATION
LRR: leucine-rich repeat
LYK4: LYSM RECEPTOR KINASE 4
MAKR: MEMBRANE ASSOCIATED KINASE REGULATOR
MAPK: mitogen-activated protein kinase
NFR1: NOD FACTOR RECEPTOR 1
NIK1: NUCLEAR SHUTTLE PROTEIN-INTERACTING RECEPTOR KINASE 1
OG: oligogalacturonides
PAMPs: pathogen-associated molecular patterns
PLL4: POL-LIKE 4
PM: plasma membrane
POL: POLTERGEIST
PRRs: pattern-recognition receptors
PTM: post-translational modification
PUB: PLANT U-BOX
RALF: RAPID ALKALINIZATION FACTOR
RBOHD: RESPIRATORY BURST OXIDASE HOMOLOG D
RHA3A: RING-H2-FINGER A 3A
RKs: receptor kinases
RLCK: receptor-like cytoplasmic kinase
RLPs: receptor-like proteins
ROS: reactive oxygen species
RTKs: receptor tyrosine kinases
SCR: S-locus cysteine-rich protein
SERKs: SOMATIC EMBRYOGENESIS RECEPTOR KINASEs
SGN3: SCHENGEN 3
SSP: SHORT SUSPENSSOR
TIR: TOLL-INTERLEUKIN RECEPTOR
TLRs: Toll-like receptors
TMK1: TRANSMEMBRANE KINASE 1
TPR: TETRATRICOPEPTIDE REPEAT
WAKs: WALL-ASSOCIATED KINASES
